# Avelumab-Induced Ocular Myasthenia Gravis: A Case Report

**DOI:** 10.7759/cureus.82958

**Published:** 2025-04-24

**Authors:** Rinrada Khaokong, Yit Ting Ling, Thangarajah Mugunthan

**Affiliations:** 1 Oncology, Royal Derby Hospital, Derby, GBR

**Keywords:** immune checkpoint inhibitor adverse effects, immunotherapy-related adverse events, myasthenia gravis (mg), neurologic complication of cancer or immunotherapy (cart), ocular myasthenia gravis, renal cell carcinoma

## Abstract

Avelumab, a programmed death-ligand 1 (PD-L1) inhibitor, has shown efficacy in renal cell carcinoma (RCC) but is associated with immune-related adverse events (irAEs), including rare neurological complications such as myasthenia gravis (MG). We report a case of a 75-year-old male patient with metastatic RCC receiving avelumab and axitinib who developed bilateral ptosis and ophthalmoplegia. Notably, there were no swallowing difficulties, limb weakness, or sensory deficits. The patient was treated with pyridostigmine and a weaning course of steroids per neurology input. Avelumab was eventually stopped due to the recurrence of symptoms. This case highlights the importance of early recognition and management of immune checkpoint inhibitor-induced MG to balance oncologic benefits with potential toxicities while also underlining the value of a multidisciplinary team (MDT) approach.

## Introduction

Immune checkpoint inhibitors (ICIs), such as avelumab, have revolutionised cancer treatment, offering durable responses in a variety of malignancies, including metastatic renal cell carcinoma (RCC). Avelumab, a programmed death-ligand 1 (PD-L1) inhibitor, enhances T-cell-mediated antitumour activity but is associated with immune-related adverse events (irAEs) due to immune system dysregulation [1,2]. Among these, neurological irAEs, particularly myasthenia gravis (MG), are rare but potentially life-threatening complications [3].

At the same time, RCCs are frequently detected incidentally during routine ultrasound or computed tomography (CT) scans, as the classic triad of flank pain, gross haematuria, and a palpable abdominal mass is observed in only 6%-10% of cases [4]. This incidental nature of diagnosis often leads to earlier detection and intervention, making ICIs a viable treatment option.

However, the emergence of MG, a rare autoimmune disorder characterised by impaired neuromuscular transmission, poses a unique clinical challenge in the use of ICI. Common manifestations include ptosis, diplopia, and generalised muscle weakness [5]. The diagnosis of ICI-induced MG is particularly challenging due to its rarity and the nonspecific nature of symptoms, which often overlap with other neurological conditions. Consequently, early recognition and prompt intervention are critical to mitigate morbidity while maintaining the efficacy of cancer treatment.

This report details the clinical presentation, diagnostic approach, and management of a patient with metastatic RCC who developed avelumab-induced MG, emphasising the importance of multidisciplinary care in addressing this rare complication.

## Case presentation

A 75-year-old male patient with a history of metastatic RCC presented to the oncology clinic with complaints of bilateral ptosis 10 days following cycle two of avelumab and axitinib. Initially, the patient experienced dry eyes with drooping of his right eye, which subsequently progressed to involve the left eye with double vision. Symptoms typically worsened later in the day. He denied any issues with swallowing, speech, or limb weakness.

The patient was referred to the neurology department for further evaluation after being clinically diagnosed in the oncology clinic as MG and was subsequently admitted for inpatient care. Routine haematology and biochemistry lab results were unremarkable (Table 1). On examination, he exhibited diplopia and bilateral ptosis, with greater severity on the right. There was also mild ophthalmoplegia in the right eye, with restricted abduction and adduction. Peripheral nerve examination findings were normal. A speech and language therapy (SALT) consultation was requested.

**Table 1:** Investigation profile at the time of admission eGFR: estimated glomerular filtration rate; MUSK: muscle-specific tyrosine kinase; VGCC: voltage-gated calcium channel

Blood	Results	Reference ranges
Haemoglobin	153 g/L	130-170 g/L
White cell count	8.04 x 10^9^/L	4-10 x 10^9^/L
Platelet	233 x 10^9^/L	150-410 x 10^9^/L
Neutrophil	6.16 x 10^9^/L	2-7 x 10^9^/L
C-reactive protein	3.3 mg/L	0-5 mg/L
eGFR	42 mL/min/1.73 m^2^	
T3	4.8 pmol/L	3.1-6.8 pmol/L
T4	15.5 pmol/L	12-22 pmol/L
Thyroid-stimulating hormone	0.76 mIU/L	0.3-5.5 mIU/L
Acetylcholine receptor Ab positive	Positive	
MUSK IgG Ab negative	Negative	
VGCC IgG Ab Negative	Negative	

Further diagnostic workup included nerve conduction studies and antibody testing. Anti-acetylcholine receptor antibodies were positive, supporting the diagnosis of MG, while anti-muscle-specific tyrosine kinase (anti-MUSK) and voltage-gated calcium channel (VGCC) antibodies were negative [6]. Nerve conduction studies demonstrated increased jitter and occasional conduction blocks, supporting a diagnosis of ocular MG.

**Figure 1:**
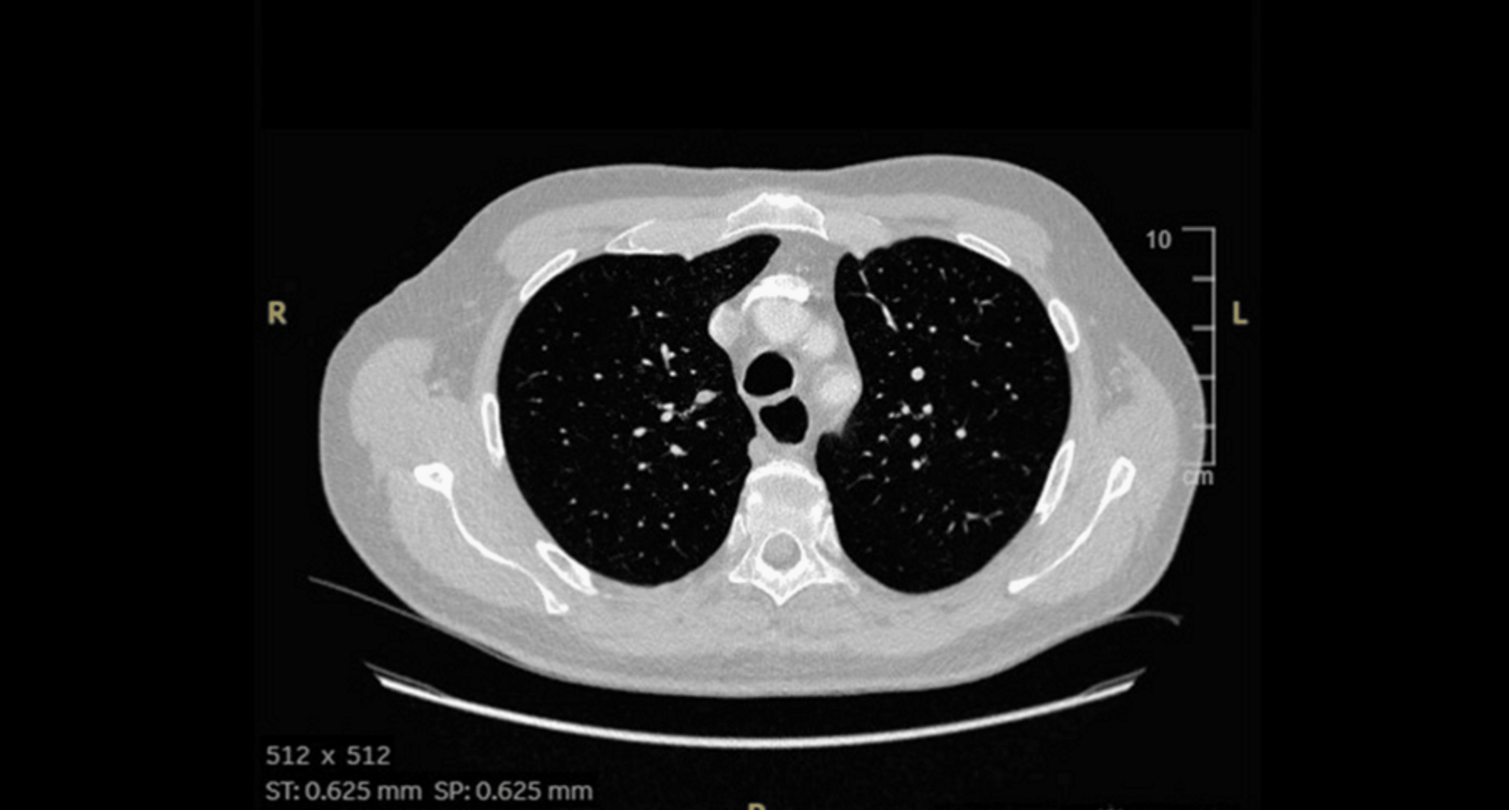
CT scan of the chest showing no thymoma CT: computed tomography

The patient was initiated on pyridostigmine and high-dose prednisolone in which he showed significant improvement in symptoms with this treatment regimen. A CT scan of the thorax was performed on admission to rule out thymoma, which returned negative (Figure 1). He was discharged with pyridostigmine and a rapid tapering regimen of prednisolone weaned by 10 mg every three days instead of the normal five-day weaning regimen to restart avelumab as soon as possible. His immunotherapy with avelumab and axitinib was resumed with close monitoring for potential adverse effects, which resulted in a relapse of his symptoms a few days later due to the rapid reduction of steroids. The patient was re-referred to the neurology team and restarted on high-dose pyridostigmine and a slower weaning regimen of prednisolone. The decision was made to stop his treatment with avelumab. Subsequently, axitinib was also stopped due to persistent proteinuria, and the patient is currently monitored with surveillance CT.

## Discussion

MG is an autoimmune disorder characterised by impaired neuromuscular transmission due to antibodies targeting acetylcholine receptors or related proteins at the neuromuscular junction. In the context of ICIs, MG likely arises due to immune dysregulation and loss of self-tolerance, leading to the activation of autoreactive T-cells [7]. In this case, the development of ocular MG after two cycles of avelumab and axitinib therapy was evidenced by bilateral ptosis and diplopia and confirmed by the presence of acetylcholine receptor antibodies and neurophysiological findings.

The rarity of avelumab-induced MG complicates its recognition and management, with fewer than 10 reported cases of its association with MG including lung adenocarcinoma and ovarian cancer, underscoring the need for heightened awareness among clinicians [8,9]. The patient's symptoms initially presented subtly, with dry eyes and right-sided ptosis, evolving to bilateral involvement.

Management of ICI-induced MG requires a delicate balance between addressing the autoimmune complication and continuing cancer treatment. This case demonstrates the importance of prompt initiation of treatment, including high-dose corticosteroids and pyridostigmine, which effectively stabilised the patient’s symptoms. Imaging ruled out thymoma, a common association with MG, while neurophysiological studies supported the diagnosis.

Immunotherapy was temporarily withheld to allow symptom stabilisation and eventually stopped when symptoms relapsed. The patient was followed up by his main oncologist with surveillance monitoring as well as neurology input if required. He also had routine telephone reviews with the immunotherapy clinical nurse specialist (CNS). This approach emphasises the need for individualised treatment strategies to maintain oncological efficacy while minimising risks associated with irAEs. Multidisciplinary collaboration involving oncologists, neurologists, and allied healthcare professionals was crucial in achieving favourable outcomes.

This case highlights the need for further research into the mechanisms underlying ICI-induced MG, optimal diagnostic pathways, evidence-based management strategies, and the importance of working with a designated specialty team to manage irAEs. Early recognition and intervention are paramount to mitigate morbidity and ensure the continuation of developing cancer therapies. This also stresses the need for follow-up with the designated immunotherapy CNS to monitor the patient for any side effects. Clinicians should maintain a high index of suspicion for MG and other neurological irAEs in patients receiving ICIs, especially those presenting with ocular or neuromuscular symptoms.

## Conclusions

This case highlights the rare but significant occurrence of avelumab-induced MG in a patient with metastatic RCC. Prompt recognition and diagnosis were facilitated by clinical examination, antibody testing, and neurophysiological studies, enabling effective management with pyridostigmine and high-dose corticosteroids. Weighing the benefits of continuing immunotherapy in RCC vs. risks of irAEs, in this case, managing MG symptoms was an important shared decision made with the patient.

The rarity of avelumab-induced MG underscores the importance of clinician awareness and multidisciplinary collaboration in addressing irAEs-related systemic disorders. This case reinforces the need for vigilance in patients receiving ICIs, particularly those presenting with neuromuscular symptoms. Early intervention with a multidisciplinary team can mitigate morbidity and support the continuation of life-prolonging therapies, balancing oncologic efficacy with patient safety. As illustrated in this case, future research should focus on refining risk stratification tools and developing clinical algorithms tailored to the early recognition of neuromuscular irAEs. In addition, clinical practice would benefit from increased awareness and education among clinicians, as well as the integration of routine neurological assessments into treatment protocols for patients receiving ICIs.
